# Development of a Gag‐Based Indirect ELISA and Its Application for Serological Surveillance of Antibodies Against Small Ruminant Lentiviruses (SRLVs)

**DOI:** 10.1155/tbed/8559688

**Published:** 2026-10-25

**Authors:** Xin Zhang, Lianfa Liu, Rigetu Zhao, Xiaozhou Xu, Wei Wang, Yongqiang Liu, Jiajia Deng, Lei Na, Xuhong Wang, Weijiao Sun, Yan Xue, Yanhong Qin, Guilin Liu, Xue-Feng Wang, Xiaojun Wang

**Affiliations:** ^1^ State Key Laboratory of Animal Disease Control and Prevention Harbin Veterinary Research Institute of Chinese Academy of Agricultural Sciences Harbin 150069 China; ^2^ Hulunbuir Hailar Center for Animal Disease Prevention and Control Hulunbuir 021000 China; ^3^ Chifeng Academy of Agricultural and Animal Husbandry Sciences Chifeng 024031 China; ^4^ College of Animal Husbandry and Veterinary Medicine Jiangsu Vocational College of Agriculture and Forestry Jurong 212400 China; ^5^ Hulunbuir Animal Disease Control Center Hulunbuir 021000 China; ^6^ Inner Mongolian Animal Disease Prevention and Control Center Hohhot 010010 China; ^7^ Jurong Animal Disease Prevention and Control Center Jurong 212400 China; ^8^ Rushan Xiacun Town Animal Husbandry and Veterinary Station Rushan 264500 China; ^9^ Institute of Western Agriculture Chinese Academy of Agricultural Sciences Changji 831100 China caas.cn; ^10^ China-Kazakhstan Joint Laboratory for Herbivorous Animal Disease Research Harbin Veterinary Research Institute Chinese Academy of Agricultural Sciences Harbin Heilongjiang Province 150069 China caas.cn

**Keywords:** CAEV, ELISA, MVV, serological surveillance, small ruminant, SRLV

## Abstract

Small ruminant lentiviruses (SRLVs) cause lifelong persistent infections in sheep and goats, impairing productivity and restricting livestock trade. Serological enzyme‐linked immunosorbent assay (ELISA) testing is the primary method for SRLV epidemiological monitoring, and most commercial ELISAs use capsid (CA), envelope, or a combination of both proteins as antigens. We previously developed a CA‐based indirect ELISA (iELISA) to detect SRLV antibodies. Here, we expressed full‐length recombinant SRLV Gag protein and further developed an iELISA using Gag as the coating antigen. The cut‐off value of the established Gag‐based iELISA was determined to be 0.093 based on sample‐to‐positive (S/P) ratio analysis of 100 SRLV‐negative sera. Parallel testing of 228 sera using the Gag‐iELISA, CA‐iELISA, and a commercial ELISA showed that the positive detection rate of the Gag‐iELISA was 35.96 percentage points higher than that of the CA‐iELISA and 4.39 percentage points higher than that of the commercial ELISA. All samples that tested positive by either the CA‐iELISA or the commercial ELISA were also positive by the Gag‐iELISA. Samples testing positive by the Gag‐iELISA but negative by the CA‐iELISA or the commercial ELISA were confirmed as SRLV antibody‐positive by Western blot (WB). Concordance analysis of the Gag‐iELISA and commercial ELISA showed 95.61% overall agreement. Endpoint dilution assays demonstrated that the Gag‐iELISA showed twice the analytical sensitivity of the CA‐iELISA and sensitivity comparable to that of the commercial ELISA. Furthermore, testing of 761 ovine sera from Hulunbuir during 2024–2025 revealed an overall SRLV seropositivity rate of 54.80%, with regional positive rates ranging from 0.00% to 84.44%. Collectively, this Gag‐based iELISA is a sensitive and practical tool for large‐scale SRLV serological surveillance.

## 1. Introduction

Small ruminant lentiviruses (SRLVs), a collective term encompassing Maedi‐Visna virus (MVV) and caprine arthritis‐encephalitis virus (CAEV), belong to the genus *Lentivirus* within the family *Retroviridae* and form a genetically diverse lentiviral population circulating in sheep and goats, as well as certain wild ruminant species [1]. Historically, SRLV was presumed to exhibit strict host specificity, with MVV predominantly afflicting sheep and CAEV largely restricted to goats. Hence, two distinct infections, namely, Maedi‐Visna (MV) and caprine arthritis encephalitis (CAE), have been recognized. Currently, the World Organization for Animal Health (WOAH) and the Ministry of Agriculture and Rural Affairs of China still designate SRLV infection in sheep as MV and that in goats as CAE. Both MV and CAE are listed as notifiable animal diseases by the WOAH and have been classified as Class III animal epidemic diseases by the Ministry of Agriculture and Rural Affairs of China. Nevertheless, accumulating epidemiological and phylogenetic evidence has clearly demonstrated that SRLV is capable of cross‐species transmission between sheep and goats [2, 3].

Infection with SRLV is usually lifelong and persistent, leading to multiorgan chronic inflammatory pathologies such as interstitial pneumonia, nonsuppurative arthritis, encephalitis, and mastitis [4]. Beyond the compromise of animal welfare, SRLV infections impose a formidable economic burden on the livestock industry due to reduced milk yield, reduced fertility and lamb production, impaired weight gain, premature culling, and restricted international livestock trade [5–9]. Currently, infections with SRLV have been reported worldwide. Notably, Europe is the geographical area with the most extensive epidemiological investigations, and individual seroprevalence in several European countries can exceed 40%, such as in Portugal and Greece [10–12]. In recent years, some studies have reported that SRLV infection is prevalent in parts of China, and the seropositivity rate in some flocks can even reach 40% [13–17].

SRLV encodes three structural proteins (Gag, Pol, and Env) as well as three accessory proteins (Vif, Rev, and Vpr‐like). Similar to other lentiviruses, the Gag precursor polyprotein (p55) is cleaved by the viral protease into three major structural proteins in mature virions: matrix protein (MA), capsid (CA) protein (p28), and nucleocapsid protein (NC) [18]. CA is one of the most abundant structural components of virions and has been widely used as a core antigenic marker for serological detection. The Pol precursor is processed to generate viral enzymes (protease, reverse transcriptase, and integrase), and the Env precursor is proteolytically processed by cellular furin into the surface glycoprotein (SU) and transmembrane protein (TM). SU and TM interact via noncovalent binding to assemble into the mature Env complex embedded on the surface of viral particles, which are responsible for entry into host cells.

Given the lack of vaccines, rigorous diagnostic screening remains the most effective strategy for flock management and SRLV infection eradication [19]. To date, there is no universally recognized gold‐standard assay for the diagnosis of infections with SRLV. The agar gel immunodiffusion test (AGID) and the enzyme‐linked immunosorbent assay (ELISA) are the preferred methods recommended by the WOAH [20] for the diagnosis of SRLV infection. AGID was once the reference assay for SRLV serological detection due to its high specificity. However, its insufficient sensitivity and the fact that results are subjectively interpreted pose notable limitations, making ELISA a dominant alternative [12, 14, 21–24]. Currently, most commercial and in‐house assays for detecting antibodies against SRLV use an indirect ELISA (iELISA) format, with CA alone or a combination of CA and Env‐derived peptides or fragments serving as antigens [25, 26]. However, low‐cost, commercially available ELISA kits are lacking in China.

Previously, our laboratory established an iELISA based on the SRLV CA protein [14]. In the present study, we further developed a novel iELISA using the full‐length SRLV Gag protein as the coating antigen for the specific detection of anti‐SRLV antibodies in serum samples. We compared the diagnostic performance of our newly developed Gag‐based iELISA with that of the previously established CA‐based iELISA and a commercial iELISA. The results confirmed that the Gag‐based iELISA exhibited the highest detection sensitivity. Subsequently, we applied this Gag‐based iELISA to test serum samples collected from Hulunbuir City, Inner Mongolia Autonomous Region, China, during 2024–2025.

## 2. Materials and Methods

### 2.1. Plasmids and Serum Samples

Three prokaryotic expression plasmids for SRLV Gag were constructed by individually cloning the full‐length Gag open reading frame (ORF) from three Chinese SRLV isolates into the pET30a (+) vector, with GenBank accession numbers MZ313871.1, KT214469.1, and MW248464.1. Herein, the pET30a‐Gag plasmid refers to the construct derived from MZ313871.1, an SRLV subtype A2 strain isolated from sheep in China in 1992 [27], and the Gag protein expressed from this plasmid was used as the detection antigen in this study. All target genes were synthesized by Ruibo Xingke Biotechnology (Beijing, China).

A total of 124 serum samples (100 SRLV‐negative and 24 SRLV‐positive), all previously verified using a commercial ELISA (ID.Vet, CVT1135T), the CA‐based iELISA, and Western blot (WB), were used to determine the cut‐off value of the Gag‐based iELISA [14]. A total of 228 serum samples, including 160 ovine sera from the Inner Mongolia Autonomous Region and 68 caprine sera from the Inner Mongolia Autonomous Region and Jiangsu Province, were used for comparative sensitivity analysis among the Gag‐based iELISA, CA‐based iELISA, and the commercial ELISA (ID.Vet, CVT1135T). A total of 761 ovine serum samples were collected from 13 county‐level administrative regions in Hulunbuir, Inner Mongolia, during 2024–2025, and all samples were tested using the Gag‐based iELISA to calculate the regional seropositivity rate of SRLV. The sampling regions included Hailar District, Genhe City, Zalantun City, Ergun City, Oroqen Autonomous Banner, Ewenki Autonomous Banner, New Barag Right Banner, Arun Banner, Morin Dawa Daur Autonomous Banner, Old Barag Banner, Yakeshi City, New Barag Left Banner, and Jalainur District (Supporting Information 1: Table S1). Sera collected in 2024 and 2025 were sourced from independent farms within each region, rather than from longitudinal sampling of the same flocks. For sampling, one township was randomly selected within each county, and 1–2 sheep farms were selected from the chosen township, with a maximum of 40 serum samples collected per farm. In addition, 325 serum samples previously collected from four provinces, namely, Xinjiang, Qinghai, Yunnan, and Fujian [14], were also assayed using the Gag‐based ELISA. This panel comprised 80 samples from Kashgar and Ili Kazakh Autonomous Prefecture (Xinjiang), 85 samples from Haixi (Qinghai), 80 samples from Kunming (Yunnan), and 80 samples from Ningde (Fujian).

### 2.2. Expression and Purification of Recombinant SRLV Gag Protein (rGag)

To obtain the rGag, the pET30a‐Gag plasmid was transformed into BL21(DE3) competent cells. The transformed cells were then cultured in a Luria‐Bertani (LB) medium supplemented with 50 μg/mL kanamycin at 37°C with shaking. When the bacterial absorbance at 600 nm (OD_600_) reached 0.6–0.8, the expression of the target protein was induced by adding isopropyl‐β‐D‐thiogalactopyranoside (IPTG; Sigma–Aldrich, St. Louis, MO, USA) to the medium to a final concentration of 0.5 mM. To establish the optimal induction conditions for efficient target protein expression, three different induction strategies were evaluated: induction at 37°C for 4 h, induction at 25°C for 10 h, and induction at 16°C for 18 h. After the induction period, bacterial pellets were collected by centrifugation at 6000 × g, washed twice with phosphate‐buffered saline (PBS), and subjected to ultrasonic lysis for 10 min to extract intracellular components containing the target protein. Finally, sodium dodecyl sulfate‐polyacrylamide gel electrophoresis (SDS‐PAGE) was performed to detect the expression level of the target protein.

Based on the optimized induction condition (16°C for 18 h with 0.5 mM IPTG), the purification of rGag was performed. Briefly, BL21(DE3) competent cells were transformed with the pET30a‐Gag plasmid. Following the induction period, bacterial pellets were collected and lysed. The supernatant was incubated with Ni‐NTA resin (GenScript, Nanjing, China) at 4°C for 1.5 h to facilitate optimal binding of rGag. After collection of the flow‐through fraction, the resin was extensively washed with 10 column volumes of wash buffer (50 mM NaH_2_PO_4_, 300 mM NaCl, and 100 mM imidazole, pH 8.0) to remove nonspecifically bound proteins. Subsequently, the target protein was eluted with four successive rounds of 2 column volumes elution buffer (50 mM NaH_2_PO_4_, 300 mM NaCl, and 500 mM imidazole). The resulting eluates were pooled and subjected to buffer exchange into a stabilization buffer consisting of 50 mM Tris‐HCl (pH 7.4), 300 mM NaCl, 10% (v/v) glycerol, and 50 mM L‐arginine. The purified rGag was analyzed by SDS‐PAGE and WB.

### 2.3. Development and Optimization of an SRLV Gag‐Based iELISA

To establish an iELISA using the purified rGag protein as the coating antigen for the specific detection of SRLV antibodies, a standard checkerboard titration method was employed to optimize the working conditions of the assay. The specific conditions were determined by selecting the parameters that yielded the maximum ratio of optical density at 450 nm (OD_450_) between the positive and negative reference sera (positive‐to‐negative [P/N] value). Initially, varying amounts of the capture antigen (50, 100, 200, and 300 ng/well) were cross‐titrated against a series of primary serum (1:50, 1:100, 1:200, and 1:400). Concurrently, the optimal working dilution for the secondary antibody (monoclonal antigoat/sheep IgG–peroxidase antibody produced in mouse, Sigma–Aldrich, USA) was evaluated at 1:5000, 1:10,000, and 1:20,000. Furthermore, we assessed the efficacy of three distinct blocking formulations (5% skim milk, 5% BSA, and 1% gelatin) alongside different blocking times (60, 90, and 120 min at 37°C). Finally, the optimal antigen‐antibody binding intervals were systematically tested (30, 60, 90, and 120 min at 37°C for primary antibody incubations, 30, 45, 60, and 90 min for secondary antibody incubations), and the optimal TMB substrate (Beyotime, China) color development time was determined by monitoring the reaction at room temperature for 5, 7, 10, and 15 min.

To standardize the assay results and minimize interplate variation, the raw OD_450_ for each well was converted into a sample‐to‐positive (S/P) ratio. This conversion was calculated using the following equation: S/P ratio = (OD_sample_‐OD_NC_)/(OD_PC_‐OD_NC_), where NC and PC represent the negative and positive controls, respectively. Subsequently, to establish a reliable diagnostic threshold, an independent validation panel comprising 100 confirmed SRLV‐negative sera and 24 known SRLV‐positive sera was evaluated. The cut‐off value was statistically defined as the mean S/P ratio of the negative sera (M) plus three standard deviations (3SD). Consequently, any serum yielding an S/P ratio greater than or equal to the calculated cut‐off value (M + 3SD) was classified as seropositive for SRLV.

### 2.4. Evaluation of the Specificity and Reproducibility of the Gag‐Based iELISA

To evaluate the analytical specificity of the developed iELISA, cross‐reactivity was assessed using a panel of six antisera against *Brucella melitensis* (*B. melitensis*), peste des petits ruminants virus (PPRV), sheep poxvirus (SPPV), Orf virus (ORFV), and foot‐and‐mouth disease virus serotypes A and O (FMDV‐A and FMDV‐O). Detailed information on these control sera has been reported previously [14].

A well‐characterized panel consisting of four serum samples (three SRLV‐positive samples with strong, moderate, and weak reactivity, together with one SRLV‐negative control) was used to evaluate the reproducibility of the iELISA. For intra‐assay reproducibility evaluation, these samples were tested in parallel on the same plate. For interassay reproducibility, three independent trials were performed on different days, with each sample analyzed in triplicate in each run. The reproducibility of the assay was quantified by calculating intra‐ and interassay coefficients of variation (CVs). CVs were calculated as the standard deviation (SD) divided by the mean optical density value and expressed as percentages.

### 2.5. Comparison of the Gag‐Based iELISA, the CA‐Based iELISA, and the Commercial ELISA

To compare the diagnostic sensitivity of the Gag‐based iELISA, the CA‐based iELISA, and a commercial iELISA (ID.Vet, CVT1135T), a total of 228 serum samples (160 sheep and 68 goat samples) were tested in parallel according to the standard operating procedure of each assay. For samples with discrepant results among the three ELISA assays, a random subset was subjected to further validation by WB, with purified recombinant Gag and CA proteins employed as antigens. The selected serum samples were diluted at 1:100 and used as the primary antibodies, and IRDye 800CW donkey antigoat IgG (diluted at 1:5000, LI‐COR Biosciences, Lincoln, NE, USA) served as the secondary antibody. All WB experimental procedures were performed as previously described [28].

To further compare the analytical sensitivity of the Gag‐based iELISA, CA‐based iELISA, and the commercial ELISA, endpoint titration assays were performed using three representative SRLV‐positive reference sera with different seroreactivity: strongly positive, moderately positive, and weakly positive samples. Each serum sample was subjected to a two‐fold serial dilution and tested in parallel using the three ELISA assays. The S/P values were calculated for each dilution, and the endpoint titer was defined as the highest serum dilution at which the sample remained above the corresponding cut‐off value for each assay.

## 3. Results

### 3.1. Expression and Purification of the rGag

To prepare rGag, the pET30a‐Gag plasmid harboring the full‐length SRLV *gag* gene was transformed into *Escherichia coli* BL21(DE3) competent cells. Optimal rGag expression was achieved using 0.5 mM IPTG at 16°C for 18 h. SDS‐PAGE analysis verified that His‐tagged rGag was successfully expressed with an approximate molecular weight of 55 kDa (Figure 1A). WB results showed that rGag specifically reacted with anti‐His antibody and SRLV‐positive serum, respectively (Figure 1B,C) but did not react with SRLV‐negative serum (Figure 1D). Additional bands detected by the anti‐His antibody presumably originated from endogenous *E. coli* proteins with histidine‐rich stretches (Figure 1B). Nevertheless, nonspecific signals remained marginal when probed with either SRLV‐positive or ‐negative serum (Figure 1C,D).

**Figure 1:**
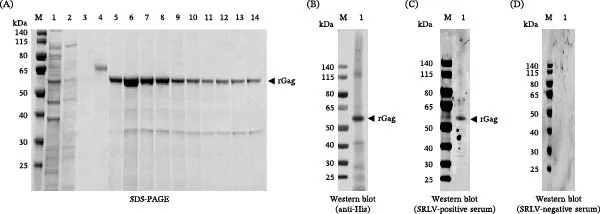
Expression and purification of recombinant SRLV Gag protein (rGag). (A) SDS‐PAGE analysis of purified His‐tagged rGag. BL21 (DE3) cells transformed with pET30a‐Gag were induced with 0.5 mM IPTG at 16°C for 18 h. The bacterial lysate supernatant was loaded onto Ni‐NTA affinity resin. After collecting the flow‐through fraction, the resin was thoroughly washed with wash buffer, and the target rGag protein was subsequently eluted. Lane M, protein molecular weight marker; lane 1, flow‐through fraction; lanes 2–3, wash fractions; and lanes 4–14, eluted protein fractions. (B) WB analysis of purified rGag protein using an anti‐His antibody. (C) WB analysis of purified rGag using SRLV‐positive serum. (D) WB analysis of purified rGag using SRLV‐negative serum. In panels B–D, lane M represents the protein molecular weight marker, and lane 1 represents purified rGag protein.

### 3.2. Establishment and Optimization of the Gag‐Based iELISA

To establish an iELISA based on rGag for the detection of antibodies against SRLV, a checkerboard titration assay was performed to determine the optimal antigen coating concentration, serum dilution, and secondary antibody dilution. According to the maximum P/N absorbance ratio, the optimal coating amount of rGag was determined to be 100 ng/well, with a serum dilution of 1:200 and a secondary antibody dilution of 1:5000 (Table 1). Blocking conditions were further optimized, and incubation with 5% skim milk at 37°C for 60 min produced the optimal blocking effect (Figure 2A). The optimal serum incubation time was 90 min at 37°C (Figure 2B), 45 min at 37°C for the secondary antibody (Figure 2C), and 7 min for the chromogenic substrate reaction time (Figure 2D).

**Figure 2:**
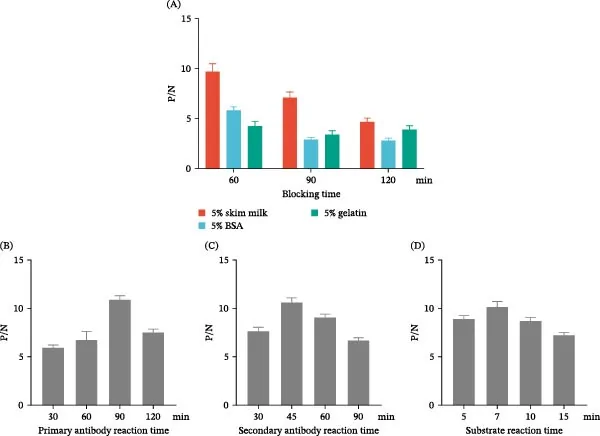
Optimization of working conditions for the Gag‐based iELISA. (A) Selection of the optimal blocking buffer and incubation time. Blocking efficiencies of three blocking reagents (5% skim milk, 5% BSA, and 1% gelatin) were compared at incubation times of 60, 90, and 120 min. (B) Determination of the optimal serum incubation time. Incubation times of 30, 60, 90, and 120 min were tested to identify the optimal serum incubation condition. (C) Optimization of incubation time for HRP‐conjugated secondary antibody. Incubation times of 30, 45, 60, and 90 min were evaluated. (D) Optimization of TMB chromogenic reaction time. Reaction times of 5, 7, 10, and 15 min were screened to obtain the highest P/N value. All data are presented as mean P/N values, and error bars denote the standard deviation (SD) of three technical replicates.

**Table 1:** Optimization of antigen coating concentration, serum dilution, and secondary antibody dilution for the Gag‐based iELISA.

Antigen coating amount (ng/well)	Secondary antibody dilution	Serum dilution											
		1:50			1:100			1:200			1:400		
		P	N	P/N	P	N	P/N	P	N	P/N	P	N	P/N
50	1:5000	1.237	0.216	5.739	1.050	0.192	5.465	0.826	0.105	7.869	0.643	0.095	6.786
	1:10,000	1.173	0.296	3.961	0.945	0.184	5.136	0.746	0.096	7.774	0.563	0.089	6.089
	1:20,000	0.935	0.235	4.008	0.935	0.192	4.90	0.654	0.085	7.698	0.482	0.086	5.629
100	1:5000	1.906	0.345	5.525	1.590	0.259	6.149	1.126	0.098	**11.496**	0.863	0.09	9.605
	1:10,000	1.623	0.337	4.837	1.346	0.26	5.201	1.025	0.109	9.386	0.774	0.094	8.219
	1:20,000	1.793	0.298	6.026	1.059	0.243	4.374	0.885	0.099	8.943	0.555	0.087	6.412
200	1:5000	2.584	0.516	5.015	1.946	0.279	6.981	1.336	0.215	6.224	1.111	0.171	6.649
	1:10,000	2.340	0.456	5.015	1.619	0.268	6.084	1.275	0.215	5.959	0.961	0.139	7.052
	1:20,000	2.055	0.46	5.143	1.343	0.264	5.115	1.044	0.258	4.076	0.781	0.099	7.970
300	1:5000	3.144	0.753	4.177	2.601	0.290	8.98	1.589	0.363	4.392	1.333	0.236	5.671
	1:10,000	2.624	0.482	5.445	2.247	0.256	8.786	1.366	0.465	2.945	1.088	0.225	4.880
	1:20,000	2.351	0.443	5.337	2.165	0.233	9.192	1.154	0.468	2.469	0.875	0.196	4.459

*Note*: The maximum P/N value is shown in bold.

### 3.3. Determination of the Cut‐Off Value for the Gag‐Based iELISA

To define a reliable diagnostic threshold for the established Gag‐based iELISA, a panel of field serum samples consisting of 100 SRLV‐negative and 24 SRLV‐positive samples (used as positive controls) was tested. The SRLV serostatus of all samples had been previously confirmed by WB, the CA‐based ELISA, and the commercial ELISA [14, 29]. Statistical analysis of the negative sera revealed a mean S/P ratio (M) of −0.000714 and a SD of 0.031238. The cut‐off value for the Gag‐based iELISA was defined as 0.093 (M + 3SD) (Figure 3A). Accordingly, samples with an S/P ratio ≥ 0.093 were considered SRLV‐positive, whereas those with an S/P ratio < 0.093 were classified as SRLV‐negative.

**Figure 3:**
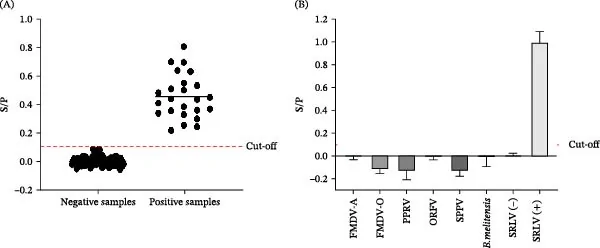
Cut‐off value determination and specificity evaluation of the Gag‐based iELISA. (A) The cut‐off value of the Gag‐iELISA was determined using 100 confirmed SRLV‐negative serum samples, with 24 confirmed positive sera used as controls. (B) The analytical specificity of the iELISA was evaluated using six antisera against common sheep and goat pathogens, including FMDV‐A, FMDV‐O, PPRV, ORFV, SPPV, and *B. melitensis*.

### 3.4. Specificity and Reproducibility of the Gag‐Based iELISA

To evaluate the specificity of the established Gag‐based iELISA, a panel of reference antisera against common ruminant pathogens, including FMDV‐A, FMDV‐O, PPRV, ORFV, SPPV, and *B. melitensis*, was tested. As illustrated in Figure 3B, the S/P ratios of all antisera against the aforementioned pathogens were well below the established cut‐off value of 0.093. In contrast, only the SRLV‐positive control serum produced a clear positive signal. These results demonstrate that the Gag‐based iELISA exhibits no cross‐reactivity with antisera against any of the tested ruminant pathogens.

To evaluate the reproducibility of the Gag‐based iELISA, three SRLV‐positive serum samples with different reactivity levels (strongly positive, moderately positive, and weakly positive) and one SRLV‐negative serum sample were tested repeatedly. As shown in Table 2, the intra‐assay CVs ranged from 1.38% to 2.38%, and the interassay CVs ranged from 1.95% to 7.91%.

**Table 2:** Assessment of the reproducibility of the Gag‐based iELISA.

Reproducibility	Serum sample	Mean ± SD	CV (%)
Intra‐assay	Strongly positive	0.9956 ± 0.0187	1.88
	Moderately positive	0.5454 ± 0.0075	1.38
	Weakly positive	0.3786 ± 0.0090	2.38
	Negative	0.1348 ± 0.0031	2.30
Interassay	Strongly positive	0.9885 ± 0.0480	4.86
	Moderately positive	0.5740 ± 0.0294	5.12
	Weakly positive	0.3541 ± 0.0280	7.91
	Negative	0.1336 ± 0.0026	1.95

### 3.5. Comparative Performance of Gag‐Based ELISA, CA‐Based ELISA, and a Commercial ELISA

A total of 228 serum samples, including 160 from sheep and 68 from goats, were subjected to parallel detection using the Gag‐based iELISA, CA‐based iELISA, and a commercial ELISA kit (ID.Vet, CVT1135T). As summarized in Figure 4A, of the 228 samples, 130 (57.02%), 48 (21.05%), and 120 (52.63%) tested positive using the Gag‐based iELISA, the CA‐based iELISA, and the commercial ELISA, respectively. Specifically, for the 160 ovine serum samples presented in Figure 4B, the number of positive samples was 86 (53.75%), 33 (20.63%), and 81 (50.63%) for the three detection methods in the above order. For the 68 caprine serum samples shown in Figure 4C, the number of positive samples was 44 (64.71%), 15 (22.06%), and 39 (57.35%) for the three detection methods in the same order. Notably, all samples that tested positive by either the CA‐based iELISA or the commercial ELISA were also positive by the Gag‐based iELISA (Supporting Information 2: Table S2). Subsequently, 40 serum samples that tested positive using the Gag‐based iELISA (30 negative in the CA‐based iELISA but positive in the commercial ELISA and 10 negative in both the CA‐based iELISA and the commercial ELISA), along with 10 samples that tested negative by all three assays, were subjected to WB analysis. The results confirmed that all Gag‐iELISA‐positive samples were WB‐positive (Supporting Information 4: Figures S1 and S2), and all negative samples in the three ELISAs were WB‐negative (Supporting Information 4: Figure S3). These findings indicate that the Gag‐based iELISA exhibits the highest positive detection rate and the lowest false‐negative rate among the three ELISA assays.

**Figure 4:**
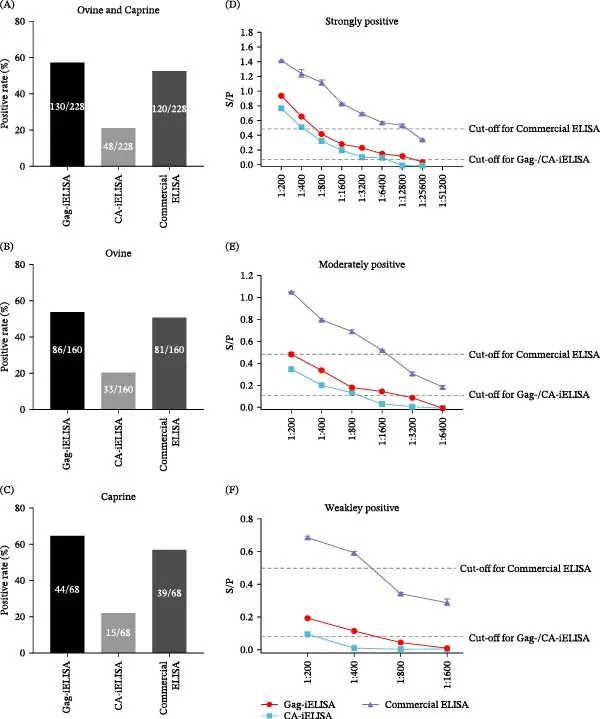
Comparative detection performance of the Gag‐based iELISA, the CA‐based iELISA, and the commercial ELISA. (A–C) Proportions of SRLV‐positive samples among 228 serum samples (160 ovine and 68 caprine sera) detected using the Gag‐based iELISA, the CA‐based iELISA, and the commercial ELISA kit. (A) Overall positive proportion of all 228 samples. (B) Positive proportion of 160 ovine samples. (C) Positive proportion of 68 caprine samples. Values inside bars indicate positive samples/total samples. (D–F) Comparison of analytical sensitivities among the Gag‐based iELISA, CA‐based iELISA, and commercial ELISA kit. Three SRLV‐positive serum samples with distinct reactivity levels, strongly positive (D), moderately positive (E), and weakly positive (F), were subjected to endpoint titration with two‐fold serial dilutions at an initial dilution of 1:200 to compare the analytical sensitivities of the three assays. Dashed lines represent the cut‐off values for each assay.

Next, a total of 325 serum samples randomly selected from an established serum bank were assayed using the Gag‐based iELISA. These samples originated from four regions: 80 ovine sera from Xinjiang, 85 ovine sera from Qinghai, 80 sera from Yunnan (predominantly caprine), and 80 sera from Fujian (predominantly caprine). Prior CA‐based iELISA screening of the serum bank yielded regional SRLV seropositivity rates of 15.38% (28/182), 7.40% (34/460), 2.61% (3/115), and 9.54% (25/262) for Xinjiang, Qinghai, Yunnan, and Fujian, respectively [14]. In the present Gag‐based iELISA testing, this sample subset yielded positive rates of 32.50% (26/80), 27.06% (23/85), 31.25% (25/80), and 28.75% (23/80), respectively (Table 3). These findings suggest that the Gag‐based iELISA has a higher detection rate for prevalent Chinese SRLV strains than the CA‐based iELISA.

**Table 3:** Seropositivity rates of SRLV antibodies detected by Gag‐iELISA and CA‐iELISA in serum samples from different regions of China.

Region	Gag‐iELISA‐positive rate (%)	CA‐iELISA‐positive rate (%)^a^
Yunnan	31.25% (25/80)	2.61% (3/115)
Fujian	28.75% (23/80)	9.54% (25/262)
Qinghai	27.06% (23/85)	7.40% (34/460)
Xinjiang	32.50% (26/80)	15.38% (28/182)

^a^The data are from Ref. [14].

Furthermore, to compare the analytical sensitivities of the Gag‐based iELISA, CA‐based iELISA, and commercial ELISA, we conducted endpoint titration experiments using three SRLV‐positive serum samples with different reactivity levels (strongly positive, moderately positive, and weakly positive). Specifically, for the strongly positive sample, the highest positive dilution was 1:12,800 for both the Gag‐based iELISA and the commercial ELISA kit, whereas the CA‐based iELISA remained positive only up to 1:6400 (Figure 4D). For the moderately positive sample, the endpoint titers of both the Gag‐based iELISA and commercial ELISA were 1:1,600, compared with 1:800 for the CA‐based iELISA (Figure 4E). For the weakly positive sample, the maximum detectable dilution for both the Gag‐based iELISA and commercial ELISA kit was 1:400, whereas the CA‐based iELISA remained positive only up to 1:200 (Figure 4F). These results indicate that the analytical sensitivity of the Gag‐based iELISA was comparable to that of the commercial ELISA and approximately two‐fold higher than that of the CA‐based iELISA.

In addition, concordance between the Gag‐based iELISA and the commercial ELISA was assessed using all 228 serum samples. As shown in Table 4, among the 228 serum samples tested in both the Gag‐based iELISA and the commercial ELISA, 120 were positive in both assays, while 98 were concordantly negative. However, 10 samples yielded discrepant results: They tested positive in the Gag‐based iELISA but negative in the commercial ELISA. Concordance analysis showed that the overall agreement was 95.61%, with a Cohen’s kappa coefficient of 0.912. The positive and negative percent agreements were 100.00% and 90.74%, respectively (Table 5).

**Table 4:** Concordance between the Gag‐based iELISA and the commercial ELISA.

	Commercial ELISA‐positive	Commercial ELISA‐negative	Total
Gag‐iELISA‐positive	120	10	130
Gag‐iELISA‐negative	0	98	98
Total	120	108	228

*Note*: Values represent the number of serum samples.

**Table 5:** Agreement analysis between the Gag‐based iELISA and the commercial ELISA.

Parameter	Value
Overall agreement	218/228 (95.61%)
Positive percent agreement	120/120 (100.00%)
Negative percent agreement	98/108 (90.74%)
Cohen’s kappa	0.912
Agreement interpretation	Almost perfect agreement

### 3.6. SRLV Antibody Detection of Field Serum Samples From Hulunbuir City

The Gag‐based iELISA developed in this study was used to detect 761 ovine serum samples collected from 13 county‐level districts of Hulunbuir, Inner Mongolia (excluding Manzhouli City), during 2024–2025 (Supporting Information 1: Table S1). As shown in Figure 5, the SRLV seropositivity rate ranged from 0.00% to 84.44% across different districts, with an overall seropositivity rate of 54.80% (417/761).

**Figure 5:**
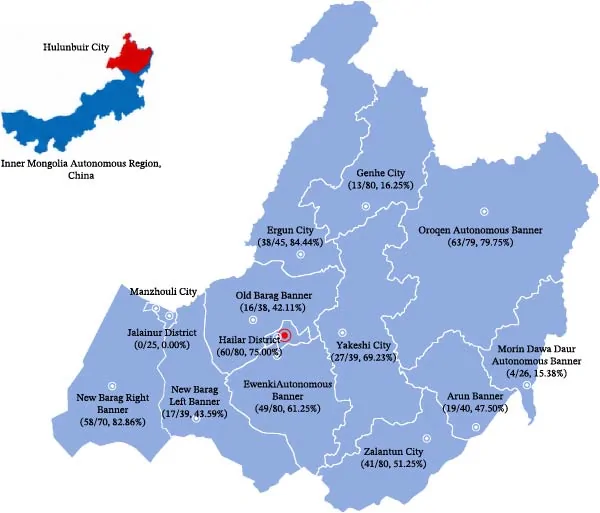
SRLV seropositivity rate across different regions of Hulunbuir based on serum samples collected in 2024–2025. A total of 761 ovine serum samples from 13 county‐level districts of Hulunbuir, Inner Mongolia Autonomous Region, China, were tested using the Gag‐based iELISA. The values in parentheses next to each geographic label indicate the number of positive samples/total number of tested samples and the corresponding regional seropositivity rate.

## 4. Discussion

SRLV exhibits high genetic diversity and are classified into five genotypes (A–E) and at least 34 subtypes. Intergenotypic nucleotide divergence ranges from 25% to 37%, whereas intragenotypic sequence variation among subtypes is 15%–27% [30]. Such extensive genetic variation poses substantial challenges to the accurate diagnosis of SRLV infection. Currently, no gold standard is available for confirming SRLV infection, and iELISA is the most widely used diagnostic assay, whose coating antigens are mainly CA alone or a combination of CA and Env epitopes [26]. In this study, the full‐length Gag protein was successfully expressed in a soluble form and subsequently used as the coating antigen to establish an iELISA. Comparative evaluation with a commercial ELISA and a CA‐based iELISA confirmed that the established Gag‐based iELISA exhibited the highest positive detection rate and the lowest false‐negative rate for both ovine and caprine sera. This assay provides a cost‐effective and reliable alternative for large‐scale serological surveillance and routine epidemiological investigation of SRLV in sheep and goats. Using this newly developed iELISA, we further conducted a large‐scale serological investigation to characterize SRLV antibody positivity among sampled sheep in Hulunbuir City, providing field serological data for the prevention and control of SRLV infection in this region.

At present, molecular epidemiological investigations of SRLV remain insufficient, and data regarding the circulating SRLV subtypes are limited in numerous regions, especially across China. A few previous studies reported that genotypes A2 and B1 are the predominant SRLV strains circulating in China [13, 15, 27, 31]. In this study, we cloned and expressed full‐length Gag proteins from three SRLV isolates recovered from Xinjiang (A2 genotype; MZ313871.1), Inner Mongolia (A2 genotype; MW248464.1), and Sichuan (B1 genotype; KT214469.1). Among these candidates, the Gag protein of the Xinjiang isolate exhibited the highest expression level (Supporting Information 4: Figure S4). Therefore, we selected this Xinjiang‐origin sequence to produce the recombinant antigen for our iELISA assay.

For lentiviruses, structural proteins Gag and Env serve as the primary targets triggering host humoral immune responses; therefore, both are commonly used as antigens in serodiagnosis [32–36]. Notably, as a group‐specific antigen, Gag possesses considerably higher sequence conservation than Env. In particular, CA, a mature cleavage subunit of the Gag polyprotein, exists in high abundance within lentiviral virions and represents the earliest viral antigen recognized by host antibodies, rendering it the predominant antigen for lentiviral serodiagnosis. Previously, we developed a CA‐based SRLV iELISA, whose analytical sensitivity was 400–600‐fold greater than that of AGID, one of the official diagnostic assays recommended by the WOAH for SRLV infection diagnosis [14]. However, this CA‐based assay showed a lower positive detection rate than the commercial SRLV ELISA (ID.Vet, CVT1135T). To overcome this limitation, herein, we developed the Gag‐based iELISA. The Gag‐based iELISA exhibited a two‐fold higher analytical sensitivity. In parallel testing of 228 serum samples, its positive detection rate was 35.96 percentage points higher than that of the CA‐based assay. In addition, 325 serum samples randomly selected from an established serum bank covering four provinces across Northwest, Southeast, and Southwest China, including Xinjiang, Qinghai, Yunnan, and Fujian, were tested using the Gag‐based iELISA. The serum bank had previously been screened by CA‐based iELISA, yielding regional SRLV seropositivity rates ranging from 2.61% to 15.38% [14]. By contrast, testing of this subset with the Gag‐based iELISA yielded positive rates ranging from 27.06% to 32.50% (Table 3). Such improved performance is presumably attributable to the expanded antigen coverage of full‐length Gag; unlike the sole CA antigen used in the previous assay, Gag consists of MA, CA, and NC domains, enabling the capture of a broader spectrum of host antibodies against SRLV and consequently elevating the field positive detection rate. This speculation is supported by previous studies, which have identified multiple epitopes within the MA, CA, and NC domains of SRLV Gag, all of which can be recognized by sera from SRLV‐infected sheep or goats [37–39]. In addition, although CA is widely recognized as a highly immunogenic component of SRLV, extensive sequence heterogeneity of the CA protein across distinct SRLV subtypes renders detection relying solely on this single antigen prone to weak signal intensities or false‐negative results arising from epitope mismatches [40]. This limitation may partly explain the discrepant samples that were negative by the CA‐based iELISA but positive by the Gag‐based iELISA, particularly those with weak or borderline reactivity in the CA‐based assay (Supporting Information 2: Table S2).

To overcome the drawbacks associated with using CA alone as the coating antigen, combinations of CA and Env‐derived antigens have been widely adopted in numerous commercial ELISAs for detecting anti‐SRLV antibodies, effectively improving the overall assay sensitivity [36]. The underlying mechanism stems from divergent seroconversion kinetics: Anti‐CA antibodies emerge during the early phase of SRLV infection, whereas anti‐Env antibodies predominate in late‐stage SRLV infection [19]. However, owing to poor recombinant expression yields of full‐length Env protein, most commercially available kits employ combined antigens consisting of CA paired with either Env subunits or SU/TM epitopes [19, 26]. In this study, the analytical sensitivity of our Gag‐based iELISA was equivalent to that of the commercial ID.Vet ELISA kit (Cat. Number CVT1135T), which uses a mixture of CA proteins and peptides derived from the SU and TM as coating antigens. When tested with 228 field serum samples, our assay exhibited a higher positive detection rate relative to the commercial kit: 130 out of 228 samples tested positive with our method, compared with 120/228 for the commercial ELISA. Importantly, all samples positive by the commercial ELISA were also positive in our Gag‐based iELISA; meanwhile, 10 samples that tested positive exclusively with our Gag‐based iELISA but negative by the commercial ELISA were further confirmed as SRLV antibody‐positive by WB, indicating that the Gag‐based iELISA has higher detection sensitivity. Although our assay uses full‐length intact Gag alone as the coating antigen and lacks Env‐derived epitopes, its diagnostic performance was comparable to that of the multiantigen commercial ELISA and showed a higher positive detection rate in this sample panel. Nevertheless, the parallel comparison in the present study only relied on 228 field serum samples; further evaluations with expanded serum panels covering animals infected with diverse SRLV genotypes are required to thoroughly compare the performance of the two assays and assess the impact of SRLV genetic variability on both assays [41]. Although the infection stages of the field animals included in this study were unknown, these findings support the robust diagnostic capability of Gag for serological screening of anti‐SRLV antibodies. Notably, while the seroconversion kinetics of anti‐SRLV CA and Env antibodies have been well characterized, the antibody dynamics targeting SRLV MA and NC remain poorly investigated to date [19, 42–44]. Characterizing the seroconversion kinetics of anti‐SRLV MA and NC antibodies may help explain the superior diagnostic performance of Gag as a screening antigen. We also cannot exclude the possibility that low sequence homology between the antigens of the commercial ELISA and circulating Chinese SRLV strains might have contributed to its lower positive detection rate.

It should be noted that the detection antigen employed in this study was derived from an SRLV subtype A2 strain isolated from sheep in China, and its diagnostic sensitivity to infections with other SRLV subtypes remains unknown. The published literature indicates that the predominant circulating strains in China are subtype A2 in sheep and subtype B1 in goats [13, 15, 27, 31]. The full‐length Gag protein of MZ313871.1 shared 91.50%–99.10% amino acid sequence identity with the examined MVV‐like strains and 74.30%–75.40% identity with the examined CAEV‐like strains (Supporting Information 3: Table S3). Although our results show that this assay yields higher positive detection rates for both ovine and caprine sera collected from multiple provinces in China compared with commercial kits and the CA‐based iELISA (Figure 4A–C; Table 3), further studies using additional sera from small ruminants with genetically subtyped SRLV infections covering diverse subtypes are required to evaluate the detection spectrum of this Gag‐based ELISA.

Previous studies have reported the prevalence of SRLV infection in some regions of China, with the individual‐level infection rate reaching up to 50.00% in certain farms [13–15, 27]. In the present study, 761 ovine serum samples obtained from Hulunbuir, Inner Mongolia, during 2024–2025 were assayed using the Gag‐based iELISA, yielding an overall SRLV seropositivity rate of 54.80%. Although the seropositivity rates varied markedly across different districts and counties, ranging from 0.00% to 84.44%, this disparity may be partly attributable to differences in sample size and sampling coverage among regions. Notably, samples from each region were collected from disparate farms across 2024–2025 rather than from a single farm. Therefore, temporal shifts in seropositivity over the 2‐year period cannot be interpreted as real‐time fluctuations in local infection rates; instead, these observations largely reflect seropositivity rate heterogeneity among distinct flocks rather than true temporal variation. Collectively, our results demonstrate a high proportion of SRLV‐seropositive animals among the sampled sheep in Hulunbuir. Nevertheless, this study has certain limitations, as detailed epidemiological data such as animal age, sex, and breed information were not collected. Further systematic epidemiological surveys and genetic subtyping of local SRLV strains are therefore warranted.

## 5. Conclusion

In summary, we have successfully established a Gag‐based iELISA for the serological detection of anti‐SRLV antibodies in sheep and goat sera. This assay showed excellent specificity, sensitivity, and reproducibility. Parallel diagnostic comparisons confirmed that the Gag‐based iELISA exhibited superior performance to the CA‐based iELISA developed in our earlier work, with diagnostic performance comparable to or even slightly exceeding that of a commercial ELISA that uses CA alongside SU‐ and TM‐derived peptides as coating antigens. Therefore, this newly developed method represents a valuable tool for large‐scale serological screening and epidemiological surveillance of SRLV.

## Author Contributions

Xin Zhang designed the study, performed the experiments, analyzed the data, and wrote the original draft. Lianfa Liu contributed to conceptualization, data analysis, and constructive discussions. Rigetu Zhao, Xiaozhou Xu, Wei Wang, Yongqiang Liu, Jiajia Deng, Lei Na, Xuhong Wang, Weijiao Sun, Yan Xue, Yanhong Qin, and Guilin Liu collected field samples. Xue‐Feng Wang, Xiaojun Wang, and Guilin Liu contributed to conceptualization and study design. Xue‐Feng Wang contributed to data analysis, manuscript drafting, review, and editing. Xiaojun Wang contributed to project administration and funding acquisition. Xin Zhang and Lianfa Liu share first authorship. Xiaojun Wang, Xue‐Feng Wang, and Guilin Liu contributed equally as corresponding authors.

## Funding

This study was supported by the Xinjiang Talent Development Fund (Grant ZZYD2023010) and the Tianchi Talent Introduction Plan (Grant IWA2023).

## Disclosure

All authors have reviewed and approved the final version of the manuscript and agree to be accountable for the content and conclusions presented in this article.

## Ethics Statement

No animal or human experiments were conducted in this study.

## Conflicts of Interest

The authors declare no conflicts of interest.

## Supporting Information

Additional supporting information can be found online in the Supporting Information section.

## Supporting information


**Supporting Information 1** Table S1: SRLV seropositivity rate across different regions of Hulunbuir based on serum samples collected in 2024–2025.

**Supporting Information 2** Table S2: The Gag‐based iELISA, the CA‐based iELISA, and the commercial ELISA test results for 228 field sera (160 sheep sera in the “Sheep” sheet and 68 goat sera in the “Goat” sheet).

**Supporting Information 3** Table S3: Full‐length Gag amino acid sequence identity between Xinjiang SRLV isolate (MZ313871.1) and representative Chinese SRLV strains.

**Supporting Information 4** Figure S1: WB analysis of serum samples that were positive by the Gag‐based iELISA but negative by both the CA‐based iELISA and commercial ELISA. Ten serum samples that tested positive by the Gag‐based iELISA but negative by both the CA‐based iELISA and commercial ELISA were further analyzed by WB. In each blot, purified recombinant SRLV Gag protein and recombinant CA protein were separated by SDS‐PAGE and transferred onto membranes, followed by incubation with the corresponding serum sample as the primary antibody (diluted at 1:100), and IRDye 800CW donkey anti‐goat IgG secondary antibody (diluted at 1:5000, LI‐COR Biosciences, Lincoln, NE, USA) served as the secondary antibody. Lane M, protein molecular weight marker; lane 1, recombinant SRLV Gag protein; and lane 2, recombinant CA protein. The numbers below each blot indicate the corresponding serum sample IDs. The arrow indicates therecombinant SRLV Gag protein (rGa). Figure S2: WB analysis of Gag‐based iELISA‐positive/CA‐based iELISA‐negative serum samples. Thirty serum samples that tested positive by the Gag‐based iELISA but negative by the CA‐based iELISA were further analyzed by WB using recombinant SRLV Gag and CA proteins as antigens. For each blot, the corresponding serum sample was used as the primary antibody (diluted at 1:100), and IRDye 800CW donkey anti‐goat IgG secondary antibody (diluted at 1:5000, LI‐COR Biosciences, Lincoln, NE, USA) served as the secondary antibody. Lane M, protein molecular weight marker; lane 1, recombinant SRLV Gag protein; and lane 2, recombinant CA protein. The numbers below each blot indicate the corresponding serum sample IDs. The arrows indicate the recombinant SRLV Gag protein (rGag) and recombinant CA protein (rCA). Figure S3: WB analysis of serum samples negative by all three ELISA assays. Ten serum samples that tested negative by the Gag‐based iELISA, CA‐based iELISA, and commercial ELISA, together with one serum sample that tested positive by all three assays (as a positive control), were further analyzed by WB using recombinant SRLV Gag and CA proteins as antigens. For each blot, the corresponding serum sample was used as the primary antibody (diluted at 1:100), and IRDye 800CW donkey anti‐goat IgG secondary antibody (diluted at 1:5000, LI‐COR Biosciences, Lincoln, NE, USA) served as the secondary antibody. Lane M, protein molecular weight marker; lane 1, recombinant SRLV Gag protein; and lane 2, recombinant CA protein. The numbers below each blot indicate the corresponding serum sample IDs. Figure S4: SDS‐PAGE analysis of prokaryotically expressed recombinant SRLV Gag proteins derived from SRLV isolates of Sichuan, Xinjiang, and Inner Mongolia. The prokaryotic expression constructs encoding Gag from the Sichuan (KT214469.1), Xinjiang (MZ313871.1), and Inner Mongolia (MW248464.1) SRLV isolates were separately transformed into BL21(DE3) competent cells, followed by induction and sample processing as described in Section 2.2 of the Materials and Methods. After cell lysis and centrifugation, soluble and insoluble fractions were analyzed separately. Lane M, protein molecular weight marker; lanes 1 and 2, soluble and insoluble fractions of the Gag protein from the Sichuan SRLV isolate, respectively; lanes 3 and 4, soluble and insoluble fractions of the Gag protein from the Xinjiang SRLV isolate, respectively; and lanes 5 and 6, soluble and insoluble fractions of the Gag protein from the Inner Mongolia SRLV isolate, respectively. The arrow indicates the recombinant SRLV Gag protein (rGag).

## Data Availability

The data supporting the findings of this study are available in the supporting information of this article.

## References

[1] Zanoni R. G. , Phylogenetic Analysis of Small Ruminant Lentiviruses, Journal of General Virology. (1998) 79, no. 8, 1951–1961, 10.1099/0022-1317-79-8-1951. 9714243

[2] Olech M. , Valas S. , Kuźmak J. , and Roques P. , Epidemiological Survey in Single-Species Flocks From Poland Reveals Expanded Genetic and Antigenic Diversity of Small Ruminant Lentiviruses, PLOS ONE. (2018) 13, no. 3, 10.1371/journal.pone.0193892, e0193892. PMC5837103 29505612

[3] Leroux C. , Chastang J. , Greenland T. , and Mornex J. F. , Genomic Heterogeneity of Small Ruminant Lentiviruses: Existence of Heterogeneous Populations in Sheep and of the Same Lentiviral Genotypes in Sheep and Goats, Archives of Virology. (1997) 142, no. 6, 1125–1137, 10.1007/s007050050147. 9229003

[4] Blacklaws B. A. , Small Ruminant Lentiviruses: Immunopathogenesis of Visna-Maedi and Caprine Arthritis and Encephalitis Virus, Comparative Immunology, Microbiology and Infectious Diseases. (2012) 35, no. 3, 259–269, 10.1016/j.cimid.2011.12.003. 22237012

[5] Peterhans E. , Greenland T. , and Badiola J. , et al.Routes of Transmission and Consequences of Small Ruminant Lentiviruses (SRLVs) Infection and Eradication Schemes, Veterinary Research. (2004) 35, no. 3, 257–274, 10.1051/vetres:2004014. 15210075

[6] Keen J. E. , Hungerford L. L. , Littledike E. T. , Wittum T. E. , and Kwang J. , Effect of Ewe Ovine Lentivirus Infection on Ewe and Lamb Productivity, Preventive Veterinary Medicine. (1997) 30, no. 2, 155–169, 10.1016/S0167-5877(96)01101-4. 9234419

[7] Juste R. A. , Villoria M. , Leginagoikoa I. , Ugarte E. , and Minguijon E. , Milk Production Losses in Latxa Dairy Sheep Associated With Small Ruminant Lentivirus Infection, Preventive Veterinary Medicine. (2020) 176, 10.1016/j.prevetmed.2020.104886, 104886. 31986356

[8] Martínez-Navalón B. , Peris C. , and Gómez E. A. , et al.Quantitative Estimation of the Impact of Caprine Arthritis Encephalitis Virus Infection on Milk Production by Dairy Goats, The Veterinary Journal. (2013) 197, no. 2, 311–317, 10.1016/j.tvjl.2012.12.020. 23384438

[9] Illius A. W. , Junkuszew A. , Nazar P. , and Savill N. J. , Performance Deficits in SRLV-Infected Sheep Quantify Progressive Disease Severity, Preventive Veterinary Medicine. (2026) 253, 10.1016/j.prevetmed.2026.106878, 106878. 41946220

[10] Jacob-Ferreira J. , Coelho A. C. , Grau Vila A. , Lacasta D. , and Quintas H. , Small Ruminant Lentivirus Infection in Sheep and Goats in North Portugal: Seroprevalence and Risk Factors, Pathogens. (2023) 12, no. 6, 10.3390/pathogens12060829, 829. PMC10305588 37375519

[11] Bouzalas I. , Apostolidi E. D. , and Scalas D. , et al.A Combined Approach for the Characterization of Small Ruminant Lentivirus Strains Circulating in the Islands and Mainland of Greece, Animals (Basel). (2024) 14, no. 7, 10.3390/ani14071119, 1119. PMC11010947 38612358

[12] de Miguel R. , Arrieta M. , and Rodríguez-Largo A. , et al.Worldwide Prevalence of Small Ruminant Lentiviruses in Sheep: A Systematic Review and Meta-Analysis, Animals (Basel). (2021) 11, no. 3, 10.3390/ani11030784, 784. PMC8000744 33799908

[13] Tian Y. , Zhang H. , and Zhang Y. , et al.Detection and Phylogenetic Analysis of Caprine Arthritis Encephalitis Virus Using TaqMan-Based qPCR in Eastern China, Veterinary Sciences. (2024) 11, no. 3, 10.3390/vetsci11030138, 138. PMC10975075 38535872

[14] Ma X. , Liu Y. , and Duan B. , et al.Recombinant CA Protein-Based ELISA for Serological Detection of Small Ruminant Lentiviruses Antibodies, Transboundary and Emerging Diseases. (2025) 2025, no. 1, 10.1155/tbed/6696495, 6696495. PMC12328048 40771840

[15] Zhao L. , Zhang L. , Shi X. , Duan X. , Li H. , and Liu S. , Next-Generation Sequencing for the Genetic Characterization of Maedi/Visna Virus Isolated From the Northwest of China, Journal of Veterinary Science. (2021) 22, no. 6, 10.4142/jvs.2021.22.e66, e66. PMC8636652 34697919

[16] Zhang Y. , Li Y. , and Yang Y. , et al.Serological Investigation of Maedi-Visna Virus Infection in Some Areas of Xinjiang, Acta Veterinaria et Zootechnica Sinica. (2020) 51, no. 8, 2022–2026.

[17] Yu S. , Lin Y. , and Chuan W. , et al.Serological Investigation of Caprine Arthritis Encephalitis and Ovine Progressive Pneumoniina in 11 Provinces of China, Chinese Veterinary Science. (2018) 48, no. 1, 34–38.

[18] Olech M. and Kuźmak J. , Molecular Characterization of Small Ruminant Lentiviruses of Subtype A5 Detected in Naturally Infected but Clinically Healthy Goats of Carpathian Breed, Pathogens. (2020) 9, no. 12, 10.3390/pathogens9120992, 992. PMC7761454 33256245

[19] Olech M. , The Genetic Variability of Small-Ruminant Lentiviruses and Its Impact on Tropism, the Development of Diagnostic Tests and Vaccines and the Effectiveness of Control Programmes, Journal of Veterinary Research. (2023) 67, no. 4, 479–502, 10.2478/jvetres-2023-0064. PMC10730557 38130459

[20] WOAH , Caprine Arthritis/Encephalitis and Maedi-Visna, 2018, WOAH Terrestrial Manual-Chapter 3.8.2.

[21] Brinkhof J. and van Maanen C. , Evaluation of Five Enzyme-Linked Immunosorbent Assays and an Agar Gel Immunodiffusion Test for Detection of Antibodies to Small Ruminant Lentiviruses, Clinical and Vaccine Immunology. (2007) 14, no. 9, 1210–1214, 10.1128/CVI.00282-06. PMC2043322 17609394

[22] Michiels R. , Van Mael E. , Quinet C. , Adjadj N. R. , Cay A. B. , and De Regge N. , Comparative Analysis of Different Serological and Molecular Tests for the Detection of Small Ruminant Lentiviruses (SRLVs) in Belgian Sheep and Goats, Viruses. (2018) 10, no. 12, 10.3390/v10120696, 696. PMC6316478 30544780

[23] Aalberts M. , Peterson K. , Moll L. , Vellema P. , and van Maanen C. , Evaluation of Five SRLV ELISAs for Fitness for Purpose in Sheep and Goat Accreditation Schemes in the Netherlands, Small Ruminant Research. (2021) 202, 10.1016/j.smallrumres.2021.106452, 106452.

[24] Silva Carvalho J. , Carrillo Gaeta N. , Castro R. , Rizzo H. , Rosati S. , and Gregory L. , P-072 Comparative Study of Indirect ELISA and AGID for Serological Detection of Small Ruminant Lentiviruses in Goats Sera, Animal - Science Proceedings. (2023) 14, no. 1, 261–262, 10.1016/j.anscip.2023.01.350.

[25] Jerre A. , Nordstoga A. B. , Dean K. R. , and Holmøy I. H. , Evaluation of Three Commercial ELISA Tests for Serological Detection of Maedi-Visna Virus Using Bayesian Latent Class Analysis, Preventive Veterinary Medicine. (2022) 208, 10.1016/j.prevetmed.2022.105765, 105765. 36181748

[26] Kalogianni A. I. , Stavropoulos I. , Chaintoutis S. C. , Bossis I. , and Gelasakis A. I. , Serological, Molecular and Culture-Based Diagnosis of Lentiviral Infections in Small Ruminants, Viruses. (2021) 13, no. 9, 10.3390/v13091711, 1711. PMC8473411 34578292

[27] Wu J.-Y. , Mi X.-Y. , and Yang X.-Y. , et al.The First Genomic Analysis of Visna/Maedi Virus Isolates in China, Frontiers in Veterinary Science. (2022) 9, 10.3389/fvets.2022.846634, 846634. PMC9263623 35812856

[28] Ma X. , Gao M. , and Zhang X. , et al.Identification and Characterization of Linear Epitopes of Monoclonal Antibodies Against the Capsid Proteins of Small Ruminant Lentiviruses, Frontiers in Microbiology. (2024) 15, 10.3389/fmicb.2024.1452063, 1452063. PMC11325181 39149208

[29] Ma X. , Development and Application of an Indirect Enzyme-Linked Immunosorbent Assay for Small Ruminant Lentivirus, 2025, Chinese Academy of Agricultural Sciences Thesis.

[30] Shah C. , Böni J. C. , and Huder J. B. , et al.Phylogenetic Analysis and Reclassification of Caprine and Ovine Lentiviruses Based on 104 new Isolates: Evidence for Regular Sheep-to-Goat Transmission and Worldwide Propagation Through Livestock Trade, Virology. (2004) 319, no. 1, 12–26, 10.1016/j.virol.2003.09.047. 14967484

[31] Wang D.-F. , Yang X.-Y. , and Wei Y.-R. , et al.Genome Characterization of the Caprine Arthritis-Encephalitis Virus in China: A Retrospective Genomic Analysis of the Earliest Chinese Isolates, Journal of Integrative Agriculture. (2023) 22, no. 3, 872–880, 10.1016/j.jia.2022.08.110.

[32] Russi R. C. , Garcia L. , Cámara M. D. S. , and Soutullo A. R. , Validation of an Indirect In-House ELISA Using Synthetic Peptides to Detect Antibodies Anti-gp90 and gp45 of the Equine Infectious Anaemia Virus, Equine Veterinary Journal. (2023) 55, no. 1, 111–121, 10.1111/evj.13555. 35007356

[33] Scicluna M. T. , Autorino G. L. , and Nogarol C. , et al.Validation of an Indirect ELISA Employing a Chimeric Recombinant Gag and Env Peptide for the Serological Diagnosis of Equine Infectious Anemia, Journal of Virological Methods. (2018) 251, 111–117, 10.1016/j.jviromet.2017.10.002. 28986292

[34] Parekh B. S. , Ou C.-Y. , and Fonjungo P. N. , et al.Diagnosis of Human Immunodeficiency Virus Infection, Clinical Microbiology Reviews. (2019) 32, no. 1, 10.1128/CMR.00064-18. PMC6302353 30487166

[35] DeMartini J. C. , Halsey W. , Boshoff C. , York D. , and Howell M. D. , Comparison of a Maedi-Visna Virus CA-TM Fusion Protein ELISA With Other Assays for Detecting Sheep Infected With North American Ovine Lentivirus Strains, Veterinary Immunology and Immunopathology. (1999) 71, no. 1, 29–40, 10.1016/S0165-2427(99)00083-5. 10522784

[36] Rosati S. , Profiti M. , and Lorenzetti R. , et al.Development of Recombinant Capsid Antigen/Transmembrane Epitope Fusion Proteins for Serological Diagnosis of Animal Lentivirus Infections, Journal of Virological Methods. (2004) 121, no. 1, 73–78, 10.1016/j.jviromet.2004.06.001. 15350735

[37] Grego E. , Bertolotti L. , and Carrozza M. L. , et al.Genetic and Antigenic Characterization of the Matrix Protein of Two Genetically Distinct Ovine Lentiviruses, Veterinary Microbiology. (2005) 106, no. 3-4, 179–185, 10.1016/j.vetmic.2004.12.007. 15778023

[38] Lacerenza D. , Genovese F. , Profiti M. , Nucera D. , and Rosati S. , Characterization of an Immunodominant Epitope of Small Ruminant Lentivirus (SRLV) Nucleoprotein, Veterinary Immunology and Immunopathology. (2008) 125, no. 3-4, 361–367, 10.1016/j.vetimm.2008.05.013. 18584880

[39] Rosati S. , Mannelli A. , Merlo T. , and Ponti N. , Characterization of the Immunodominant Cross-Reacting Epitope of Visna Maedi Virus and Caprine Arthritis-Encephalitis Virus Capsid Antigen, Virus Research. (1999) 61, no. 2, 177–183, 10.1016/S0168-1702(99)00031-3. 10475088

[40] Grego E. , Profiti M. , and Giammarioli M. , et al.Genetic Heterogeneity of Small Ruminant Lentiviruses Involves Immunodominant Epitope of Capsid Antigen and Affects Sensitivity of Single-Strain-Based Immunoassay, Clinical and Vaccine Immunology. (2002) 9, no. 4, 828–832, 10.1128/CDLI.9.4.828-832.2002. PMC120019 12093681

[41] Cardinaux L. , Zahno M.-L. , Deubelbeiss M. , Zanoni R. , Vogt H.-R. , and Bertoni G. , Virological and Phylogenetic Characterization of Attenuated Small Ruminant Lentivirus Isolates Eluding Efficient Serological Detection, Veterinary Microbiology. (2013) 162, no. 2–4, 572–581, 10.1016/j.vetmic.2012.11.017. 23206411

[42] Carrozza M. L. , Mazzei M. , and Lacerenza D. , et al.Seroconversion Against SU5 Derived Synthetic Peptides in Sheep Experimentally Infected With Different SRLV Genotypes, Veterinary Microbiology. (2009) 137, no. 3-4, 369–374, 10.1016/j.vetmic.2009.01.032. 19251384

[43] Reina R. , Juganaru M. M. , and Profiti M. , et al.Immunological Parameters in Goats Experimentally Infected With SRLV Genotype E, Strain Roccaverano, Veterinary Immunology and Immunopathology. (2011) 139, no. 2–4, 237–244, 10.1016/j.vetimm.2010.11.001. 21122927

[44] Lacerenza D. , Giammarioli M. , and Grego E. , et al.Antibody Response in Sheep Experimentally Infected With Different Small Ruminant Lentivirus Genotypes, Veterinary Immunology and Immunopathology. (2006) 112, no. 3-4, 264–271, 10.1016/j.vetimm.2006.03.016. 16698088

