# Supplementary material for: Development of a Gag‐Based Indirect ELISA and Its Application for Serological Surveillance of Antibodies Against Small Ruminant Lentiviruses (SRLVs)
Source: Transbound Emerg Dis. 2026 Oct 25;2026:8559688. doi: 10.1155/tbed/8559688 (PMC13695883; doi:10.1155/tbed/8559688)
Supplement: Supplementary file 1 [file TBED-2026-8559688-s001.docx]

**Table S3.** Full-length Gag amino acid sequence identity between Xinjiang SRLV isolate (MZ313871.1) and representative Chinese SRLV strains.

| Accession No. | Subgenotype | Host species | Isolation region | Identity with MZ313871.1 (%) |
| --- | --- | --- | --- | --- |
| KR011757.1 | Not defined | Sheep | Sichuan | 99.10 |
| MW248464.1 | A2 | Sheep | Inner Mongolia | 91.50 |
| MZ313872.1 | A2 | Sheep | Xinjiang | 99.10 |
| PP854830.1 | A2 | Sheep | China | 98.20 |
| AY900630.1 | Not defined | Goat | Gansu | 74.70 |
| GQ996380.1 | Not defined | Goat | Shanxi | 74.50 |
| GU120138.1 | Not defined | Goat | Shanxi | 74.30 |
| KT214469.1 | B1 | Goat | Sichuan | 75.40 |
| KT749878.1 | B1 | Goat | Shandong | 75.20 |
| KT749879.1 | B1 | Goat | Shaanxi | 75.40 |
| KT749880.1 | B1 | Goat | Guizhou | 75.40 |
| KT749881.1 | B1 | Goat | Gansu | 75.40 |

Notes: Accession No. denotes the GenBank accession number of Chinese SRLV strains; "Not defined" means no explicit subgenotype designation was provided from the accessible sequence-associated source.
