# Supplementary material for: Development of a Gag‐Based Indirect ELISA and Its Application for Serological Surveillance of Antibodies Against Small Ruminant Lentiviruses (SRLVs)
Source: Transbound Emerg Dis. 2026 Oct 25;2026:8559688. doi: 10.1155/tbed/8559688 (PMC13695883; doi:10.1155/tbed/8559688)
Supplement: Supplementary file 2 [file TBED-2026-8559688-s003.docx]

**Table S1.** SRLV seropositivity rate across different regions of Hulunbuir based on serum samples collected in 2024–2025

| Area | Year | Positive rate | Year | Positive rate |
| --- | --- | --- | --- | --- |
| Hailar District | 2024 | 97.50% (39/40) | 2025 | 52.50% (21/40) |
| Genhe City | 2024 | 12.50% (5/40) | 2025 | 20.00% (8/40) |
| Zalantun City | 2024 | 75.00% (30/40) | 2025 | 27.50% (11/40) |
| Oroqen Autonomous Banner | 2024 | 77.50% (31/40) | 2025 | 82.05% (32/39) |
| Ewenki Autonomous Banner | 2024 | 42.50% (17/40) | 2025 | 80.00% (32/40) |
| New Barag Right Banner | 2024 | 77.42% (24/31) | 2025 | 87.18% (34/39) |
| Arun Banner | 2024 | 47.50% (19/40) | 2025 | — |
| Morin Dawa Daur Autonomous Banner | 2024 | 15.38% (4/26) | 2025 | — |
| Old Barag Banner | 2024 | 42.11% (16/38) | 2025 | — |
| Ergun City | 2024 | 80.00% (4/5) | 2025 | 85.00% (34/40) |
| Yakeshi City | 2024 | — | 2025 | 69.23% (27/39) |
| New Barag Left Banner | 2024 | — | 2025 | 43.59% (17/39) |
| Jalainur District | 2024 | — | 2025 | 0.00% (0/25) |
| Unrecorded regions | 2024 | 30.00% (12/40) | 2025 | — |
| Total | 2024 | 52.89% (201/380) | 2025 | 56.69% (216/381) |

Note: “—” indicates that no samples were collected from the corresponding region in that year.
