# Supplementary material for: Development of a Gag‐Based Indirect ELISA and Its Application for Serological Surveillance of Antibodies Against Small Ruminant Lentiviruses (SRLVs)
Source: Transbound Emerg Dis. 2026 Oct 25;2026:8559688. doi: 10.1155/tbed/8559688 (PMC13695883; doi:10.1155/tbed/8559688)
Supplement: Supplementary file 3 [file TBED-2026-8559688-s004.docx]

Development and Application of a Gag-Based Indirect ELISA for the Detection of Small Ruminant Lentivirus (SRLV) Antibodies

Xin Zhang^1#^, Lianfa Liu^2#^, Rigetu Zhao^3^, Xiaozhou Xu^4^, Wei Wang^5^ , Yongqiang Liu^6^ , Jiajia Deng^7^ , Lei Na^4^, Xuhong Wang^6^, Weijiao Sun^5^, Yan Xue^5^, Yanhong Qin^8^, Guilin Liu^5*^, Xue-Feng Wang^1*^, Xiaojun Wang^1,9,10*^

**^Supplementary materials^**

**Figure S1. WB analysis of serum samples that were positive by the Gag-based iELISA but negative by both the CA-based iELISA and commercial ELISA.** Ten serum samples that tested positive by the Gag-based iELISA but negative by both the CA-based iELISA and commercial ELISA were further analyzed by WB. In each blot, purified recombinant Gag protein and recombinant CA protein were separated by SDS-PAGE and transferred onto membranes, followed by incubation with the corresponding serum sample as the primary antibody (diluted at 1:100), and IRDye 800CW donkey anti-goat IgG secondary antibody (diluted at 1:5000, LI-COR Biosciences, Lincoln, NE, USA) served as the secondary antibody. Lane M, protein molecular weight marker; lane 1, recombinant Gag protein; lane 2, recombinant CA protein. The numbers below each blot indicate the corresponding serum sample IDs. The arrow indicates the recombinant Gag protein (rGag).

**Figure S2.** **WB analysis of Gag-based iELISA-positive/CA-based iELISA-negative serum samples**. Thirty serum samples that tested positive by the Gag-based iELISA but negative by the CA-based iELISA were further analyzed by WB using recombinant Gag and CA proteins as antigens. For each blot, the corresponding serum sample was used as the primary antibody (diluted at 1:100), and IRDye 800CW donkey anti-goat IgG secondary antibody (diluted at 1:5000, LI-COR Biosciences, Lincoln, NE, USA) served as the secondary antibody. Lane M, protein molecular weight marker; lane 1, recombinant Gag protein; lane 2, recombinant CA protein. The numbers below each blot indicate the corresponding serum sample IDs. The arrows indicate the recombinant Gag protein (rGag) and recombinant CA protein (rCA).

**Figure S3**. **WB analysis of serum samples negative by all three ELISA assays**. Ten serum samples that tested negative by the Gag-based iELISA, CA-based iELISA, and commercial ELISA, together with one serum sample (N15) that tested positive by all three assays (as a positive control), were further analyzed by WB using recombinant Gag and CA proteins as antigens. For each blot, the corresponding serum sample was used as the primary antibody (diluted at 1:100), and IRDye 800CW donkey anti-goat IgG secondary antibody (diluted at 1:5000, LI-COR Biosciences, Lincoln, NE, USA) served as the secondary antibody. Lane M, protein molecular weight marker; lane 1, recombinant Gag protein; lane 2, recombinant CA protein. The numbers below each blot indicate the corresponding serum sample IDs.

.

**Figure S4. SDS-PAGE analysis of prokaryotically expressed recombinant Gag proteins derived from SRLV isolates of Sichuan, Xinjiang, and Inner Mongolia.** The prokaryotic expression constructs encoding Gag from the Sichuan (KT214469.1), Xinjiang (MZ313871.1), and Inner Mongolia (MW248464.1) SRLV isolates were separately transformed into BL21(DE3) competent cells, followed by induction and sample processing as described in Section 2.2 of the Materials and Methods. After cell lysis and centrifugation, soluble and insoluble fractions were analyzed separately. Lane M, protein molecular weight marker; lanes 1 and 2, soluble and insoluble fractions of the Gag protein from the Sichuan SRLV isolate, respectively; lanes 3 and 4, soluble and insoluble fractions of the Gag protein from the Xinjiang SRLV isolate, respectively; lanes 5 and 6, soluble and insoluble fractions of the Gag protein from the Inner Mongolia SRLV isolate, respectively. The arrow indicates the recombinant Gag protein (rGag).
